# Outcome analysis of shunt surgery in hydrocephalus

**DOI:** 10.4103/0971-9261.57700

**Published:** 2009

**Authors:** Ashraf Ahmed, Gursev Sandlas, Paras Kothari, Dinesh Sarda, Abhaya Gupta, Parag Karkera, Prashant Joshi

**Affiliations:** Department of Pediatric Surgery, Lokmanya Tilak Muncipal General Hospital, Sion, Mumbai, India

**Keywords:** Complications, hydrocephalus, outcome, shunt surgery, shunt revision

## Abstract

**Aim:**

To study the clinical outcome of shunt surgeries in children suffering from hydrocephalus.

**Methods:**

A prospective study of 50 children with hydrocephalus who underwent a ventriculo-peritoneal shunt insertion over a period of two years. These patients were then followed up for shunt related complications, shunt revisions and outcome.

**Results:**

Twenty six of the 50 patients (52%) suffered from complications. The most common complications were shunt blockage (n=7) and shunt infection (n=6). These complications necessitated repeated shunt revisions.

**Conclusions:**

Infective complications of hydrocephalus are more likely to leave behind an adverse neurological outcome in the form of delayed milestones and mental retardation.

## INTRODUCTION

Hydrocephalus is one of the most common clinical conditions affecting the central nervous system with an incidence of three to four per 1000 births for congenital hydrocephalus.[1] However, with the advent of shunt surgeries, these children can be assured of a near normal neurological development. Complications of shunt surgeries requiring shunt revisions have been studied in literature.[2,3] The present study is a prospective analysis of hydrocephalic children who had undergone shunt surgeries with regard to the etiology, clinical profile and outcome.

## MATERIAL AND METHODS

This is a prospective study of 50 cases of hydrocephalus which underwent ventriculo-peritoneal (VP) shunt insertion between August 2006 and July 2008. It included. children with hydrocephalus due to congenital or acquired causes with a minimum follow-up period of 12 months. A detailed record was maintained with regard to name, age, sex, etiology, clinical features, investigations including imaging and treatment. All children underwent an initial ultrasonography (USG) of the brain (n=35) through the window of anterior fontanelle to assess ventricular dilatation and ventricular: parenchyma thickness ratio. Older or suspected meningitis patients underwent computerized tomography (CT) scan of the brain (n=15). Magnetic resonance imaging (MRI) was performed in cases where there was a structural lesion causing hydrocephalus.

The device used was Chhabra's medium pressure slit and spring valve shunt. Cerebrospinal fluid (CSF) flow in this type of shunt is pressure regulated and flow rate type. The whole shunt is radio-opaque. It is an economically viable shunt used extensively in India. Details of complications necessitating shunt revisions and outcome were maintained in the follow-up.

## RESULTS

Of the 50 patients, 28 were males and 22 females. Age at the time of shunt insertion, for the first time, ranged from one day to 7 years with majority (76%) of them being below one year. The causes of hydrocephalus were varied with the predominant cause being aqueductal stenosis and hydrocephalus associated with meningomyelocele [Table 1]. Increasing head size was a common presentation. A few of them presented with vomiting, headache, refusal to feed, fever and convulsions [Tables 2 and 3].

**Table 1:** Etiology of Hydrocephalus

Causative factor	Number of Patients
Aqueductal stenosis	17
Spinal dysraphism:	
Meningomyelocele	15
Occipital encephalocoele	1
Dandy Walker syndrome	3
Noonan Syndrome	1
Post tubercular meningitis	6
Post pyogenic meningitis	5
Intraventricular Hemorrhage	1
Post traumatic	1
Total	50

**Table 2:** Clinical presentation [symptoms]

Symptoms	Number of Patients [n]
Asymptomatic	17
Vomiting	7
Headache	8
Refusal of feeds	4
Decreased activity	2
Fever	8
Convulsions	4
Total	50

**Table 3:** Clinical Presentation [signs]

Signs	Number of patients [n]
Increased head circumference	40
Splayed cranial sutures	36
Sunset sign	15
Higher mental functions	
Ataxia	1
Hemiparesis	2
Paraparesis	8
Meningomyelocele	15
Occipital encephalocoele	1
Tense anterior fontanelle	38
Fundus examination:	
Papilloedema	9
Optic atrophy	0

**Figure 1:**
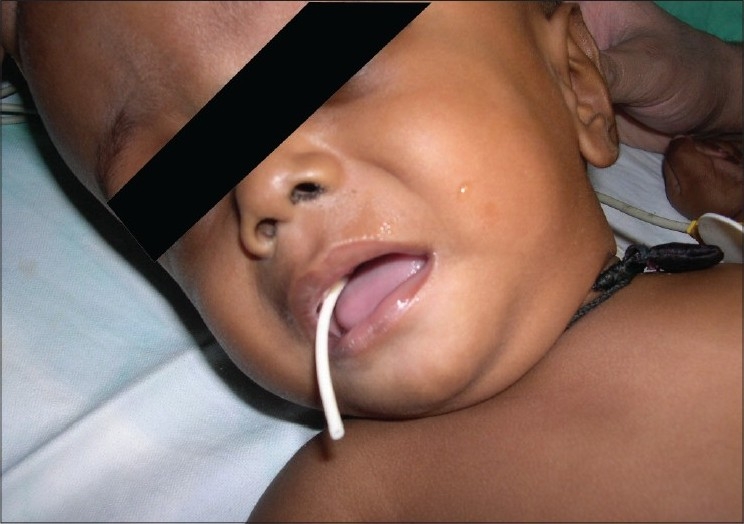
Oral Extrusion of Ventricular End of VP Shunt

**Figure 2:**
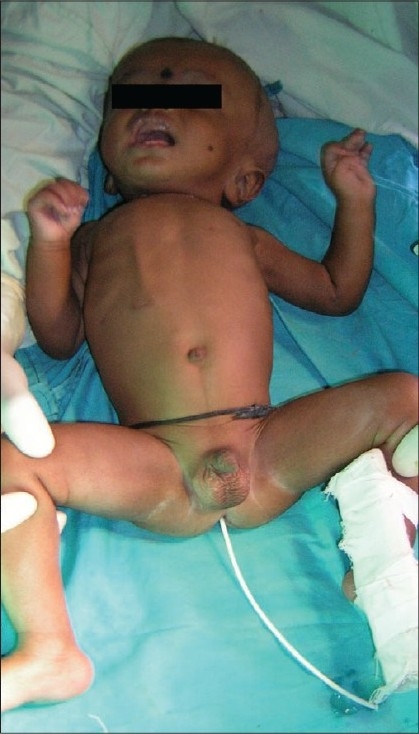
Rectal Extrusion of Peritoneal End of VP Shunt

**Figure 3:**
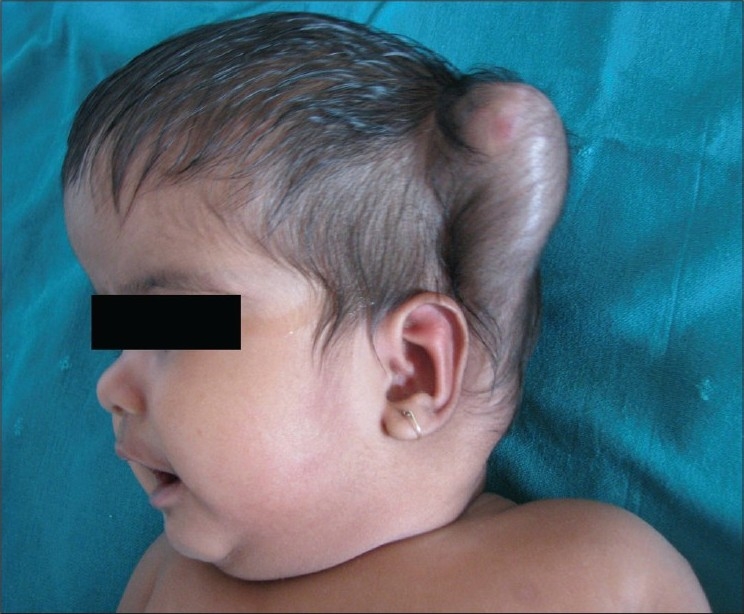
CSF Varix

Twenty six patients (52%) had complications. The most common complications[Table 4] were shunt blockage (n=7) and shunt infection (n=6). There were other complications namely shunt migration, shunt malfunction and pseudo cyst formation [Figures 1–3].

**Table 4:** Complications

Complications	Number of patients
Shunt obstruction	7 [14]
Shunt infection	6 [12]
Shunt migration:	5 [10]
Ventricular end	2
Peritoneal end	3
Shunt malfunction:	4 [8]
Ventricular end [CSF varix]	3
Peritoneal end [pseudocyst]	1
Intestinal obstruction	2 [4]
Inguinal hernia	2 [4]
Total	26 [52]

Figures in parentheses are in percentage

## DISCUSSION

Hydrocephalus is a common clinical condition[4] and majority of the patients are infants under one year as in the present study (76%). Congenital aqueductal stenosis accounts for about 10% of all hydrocephalus cases in children.[5] Aqueductal stenosis was the one of the commonest cause of hydrocephalus in our study as well (n = 17, 34%), rivaled only by spinal dysraphism (n = 16, 32%) which could probably be attributed to low socio-economic strata of the study group. In our study, hydrocephalus was detected in almost all cases of meningomyelocele. It developed either after surgical repair of the defect or was present primarily. Dandy-Walker syndrome[6,7] was present in three cases (6%).

Hydrocephalus secondary to meningitis was seen in 22% of our cases. Of these, tuberculosis accounted for 12% of cases (n = 6). Most of the patients with central nervous system tuberculosis had an identifiable systemic disease. Hydrocephalus manifested itself within four to six weeks of the disease.[8] This required anti-tubercular therapy with shunt surgery. Bacterial meningitis may cause hydrocephalus as an uncommon sequelae. In most cases, complications developed insidiously over weeks to months. We had five cases of non-tubercular meningitis causing hydrocephalus. Neurological sequelae like mental retardation were more common in the tuberculous group at follow-up.

Majority of the patients were clinically asymptomatic. Headache was present in (16%); vomiting (14%), refusal of feed (8%) and lethargy (4%). These were generally more commonly observed in infants. Increase in head circumference was the most common sign (80%) followed by tense anterior fontanelle and splayed cranial sutures (76%). Sunset sign, which probably represents upward gaze palsy due to compression or axial dislocation of the tectal region of the brainstem,[9] was seen in 30% cases. Papilloedema was seen in only nine (18%) cases which could be explained by the fact that majority (76%) of the study group were infants (< 1 yr). Neurological deficits were more common in older children with post-meningitis hydrocephalus. Kirkpatrick et al. reported 28% asymptomatic patients in shunted group and 49% incidence in non-shunted group[10]). Headache was present in 28% of 157 cases and its incidence was 33% in non-shunted group and 46% in shunted group. The most common symptoms were vomiting, behavioral changes, headache, and anorexia in decreasing order of frequency and most common sign was increasing head circumference (76%). The next common sign was a tense anterior fontanelle (65%) followed by splayed cranial sutures (39%), scalp vein distension (33%), sun setting sign (22%) and papilloedema (7%). Low incidence of papilloedema can be explained by the fact that the level of the block is at the level of ventricle or subarachnoid space. So the fluid can not reach the optic sheath and vein is not compressed.[11]

Patients with hydrocephalus are treated either with ventriculoperitoneal shunt or third ventriculostomy. All our patients underwent ventriculoperitoneal shunt at the primary presentation. Ventricular CSF shunting causes a multitude of complications, the most common is mechanical obstruction as in our study (n=7, 14%). This may occur at either or both ends, but usually due to obstruction of ventricular catheter by entrapped choroid plexus tissue, intraventricular debris or gliosis around the catheter tip. The incidence of proximal obstruction is approximately 30%. This incidence is highest in early postoperative period and decreases with time. We had proximal obstruction in six of the seven cases. Distal obstruction is due to accumulation of debris in the slit valve. The dead space beneath the slit valve favors this accumulation. Debris is composed of acellular fibrin clumps surrounded by large number of macrophages, lymphocytes and fibroblasts. We had one patient with peritoneal end blockage by tissue debris.

The second most common complication of these procedures is infection. This occurs in 5 to 10% of the cases and is usually the result of prior infection of CSF or infection introduced during surgery. Ventriculitis, meningitis and local wound infection are the most common clinical manifestations. Infection rate in the present study is 12% (n=6). Infection is generally due to organisms of low virulence, most commonly Staphylococcus epidermidis.[12] *Staphylococcus aureus*, Gram negative bacilli and mixed pathogens are the next most common agents encountered. The organism cultured in our series was predominantly the Staphylococcus species.

Migration of shunt was observed in 10% (n = 5) of cases. Two had proximal migration of the shunt into the lateral ventricle. Distal catheter had migrated and was extruded per rectally in three cases. The incidence of shunt migration is 7.5%. Shunt malfunction leading to sequelae like CSF varix, abdominal pseudo cyst was noted in eight per cent of cases (n=4). About four per cent (n=2) of the cases presented with intestinal obstruction and another four per cent (n=2) developed inguinal hernias following shunt surgery.

During a mean follow-up period of one year, 18 of the 50 cases (36%) required shunt revisions due to shunt related complications. Eight of these cases required multiple shunt revisions, mostly due to shunt infection. Shunt infection was treated by exteriorization of shunt, intermittent CSF tapping and intravenous antibiotics based on culture and sensitivity of CSF and shunt catheter. Revision shunt was done only after two consecutive CSF samples demonstrated absence of infection.

Twenty four of the 50 children (48%) had no complication following shunt insertion and improvement was noted in neurological/ functional status of the subjects at follow-up. Developmental milestones were delayed in 10 cases (20%). Five cases of post infective, two of spinal dysraphism, one of Dandy-Walker syndrome and one with intraventricular hemorrhage. Mental retardation was seen in a case of Noonan's syndrome and three cases of post infective hydrocephalus.

Hydrocephalus is a common disorder of central nervous system in children caused principally by congenital aqueductal stenosis. Infections (tubercular, pyogenic) and spinal dysraphism represent the potentially preventable causes of hydrocephalus amenable to improvements in quality of life with better outreach of public health measures in developing countries. While increase in head size is only sign of hydrocephalus among most of the children below two years, older children may present with the classical triad of headache, vomiting and papilloedema. The development of shunt surgeries has remarkably changed the outcome in these patients with better prospects of leading a normal life. However, complications like infection and obstruction warranting shunt revisions continue to adversely affect the outcome. Infective causes of hydrocephalus are also more likely to leave behind an adverse neurological outcome in the form of delayed milestones and mental retardation.
